# PDPN marks a subset of aggressive and radiation-resistant glioblastoma cells

**DOI:** 10.3389/fonc.2022.941657

**Published:** 2022-08-10

**Authors:** Aram S. Modrek, Eskil Eskilsson, Ravesanker Ezhilarasan, Qianghu Wang, Lindsey D. Goodman, Yingwen Ding, Ze-Yan Zhang, Krishna P. L. Bhat, Thanh-Thuy T. Le, Floris P. Barthel, Ming Tang, Jie Yang, Lihong Long, Joy Gumin, Frederick F. Lang, Roel G. W. Verhaak, Kenneth D. Aldape, Erik P. Sulman

**Affiliations:** ^1^ Department of Radiation Oncology, New York University (NYU) Langone School of Medicine, New York, NY, United States; ^2^ Department of Genomic Medicine, The University of Texas M.D. Anderson Cancer Center, Houston, TX, United States; ^3^ Department of Bioinformatics, Nanjing Medical University, Nanjing, Jiangsu, China; ^4^ Duncan Neurological Research Institute, Baylor College of Medicine, Houston, TX, United States; ^5^ Department of Translational Molecular Pathology, The University of Texas M.D. Anderson Cancer Center, Houston, TX, United States; ^6^ Department of Anesthesiology, University of Texas Medical School, Houston, TX, United States; ^7^ The Jackson Laboratory, Farmington, CT, United States; ^8^ Department of Neurosurgery, The University of Texas M.D. Anderson Cancer Center, Houston, TX, United States; ^9^ Center for Cancer Research, National Cancer Institute, Bethesda, MD, United States; ^10^ New York University (NYU) Langone Laura and Isaac Perlmutter Cancer Center, New York, NY, United States

**Keywords:** glioma, glioblastoma, PDPN, podoplanin, CD133, radioresistance, radiation oncology, neuro-oncology

## Abstract

Treatment-resistant glioma stem cells are thought to propagate and drive growth of malignant gliomas, but their markers and our ability to target them specifically are not well understood. We demonstrate that podoplanin (PDPN) expression is an independent prognostic marker in gliomas across multiple independent patient cohorts comprising both high- and low-grade gliomas. Knockdown of PDPN radiosensitized glioma cell lines and glioma-stem-like cells (GSCs). Clonogenic assays and xenograft experiments revealed that PDPN expression was associated with radiotherapy resistance and tumor aggressiveness. We further demonstrate that knockdown of PDPN in GSCs *in vivo* is sufficient to improve overall survival in an intracranial xenograft mouse model. PDPN therefore identifies a subset of aggressive, treatment-resistant glioma cells responsible for radiation resistance and may serve as a novel therapeutic target.

## Introduction

World Health Organization grade IV glioblastomas (GBMs) are the most frequently occurring primary malignant brain tumors in adults (1). While long-term survivors exist, few GBM patients survive beyond 2 years despite aggressive standard of care incorporating chemotherapy and radiation therapy (RT) and tumor-treating fields after maximal surgical tumor resection (2–4). The cancer stem cell theory suggests that a clonal population within the tumor has stem-cell properties, which include indefinite potential for self-renewal (5). These cancer stem cells are thought to be resistant to current cytotoxic treatment modalities and therefore responsible for GBM progression even after treatment in large part due to their plasticity and ability to repopulate the tumor (6). Singh et al. first identified such a population in GBM; they proposed that a CD133^+^ fraction of cells compromised, or partially captured, the tumor-initiating niche (7).

Expression of the type-I integral membrane glycoprotein podoplanin (PDPN), also known as OTS-8, PA2.26, gp36, gp38, RANDAM-2, T1-α, and aggrus, correlates with defining glioma stem cell characteristics (8). PDPN is expressed in some normal adult tissues, such as lymphatic endothelium, and has functionally been implicated in migration, epithelial-to-mesenchymal transition, tumor initiation in squamous cell carcinoma, and inflammation and immune evasion in glioblastoma (9–11). In contrast to CD133, PDPN exhibits a more prominent cellular distribution and has a gene expression profile that increases with glioma grade (12, 13). We hypothesize that GSC characteristics are not constrained to a small population of neoplastic CD133^+^ cells in GBM and that PDPN identifies a population of aggressive-treatment-resistant GSCs that may be contributing to poor GBM patient outcome.

In the present study, we examined PDPN expression across large glioma cohorts in addition to The Cancer Genome Atlas (TCGA) and found PDPN to indeed be an independent prognostic marker among glioma patients. We experimentally characterized the function of PDPN using patient-derived GSCs *in vitro* and *in vivo* and found that its expression is correlated with CD133 expression, but not vice versa. In xenograft tumorigenesis experiments, we discovered that PDPN expression greatly influences tumor growth.

## Materials and methods

### The cancer genome atlas cohort data

**Figure 1:**
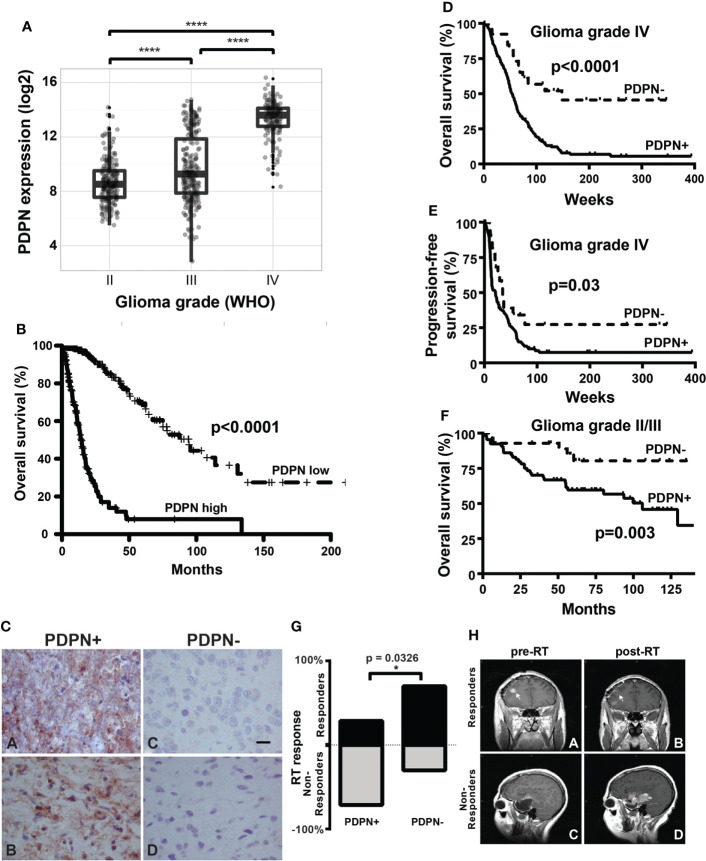
PDPN is expressed in infiltrating gliomas and negatively correlates with survival. **(A)** Boxplots show increasing PDPN expression across increasing glioma grades in TCGA (n=601, ****p<0.0001). **(B)** Kaplan–Meier curves show that PDPN mRNA expression inversely correlates with OS across glioma grades in TCGA cohorts. Median OS for patients with PDPN-high and PDPN-low tumors was 14.4 and 94.5 months, respectively (n=601, p<0.0001). **(C)** Representative IHC examples of PDPN expression in FFPE tumor tissue using the M2A antibody in tumors. Subpanels **(A, B)** shows intense cytoplasmic staining in nearly all cells, while expression is undetectable in tumors in subpanels **(C, D)**. **(D)** Kaplan–Meier curves show that PDPN protein expression inversely correlates with OS among GBM WHO grade IV patients. Median OS for PDPN+ and PDPN− groups was 53.4 and 148.1 weeks, respectively (n=206, p<0.0001). IHC KM curves are shown as solid (PDPN+) and dashed (PDPN−) lines. **(E)** Kaplan–Meier curves show that PDPN protein expression inversely correlates with PFS among GBM WHO grade IV patients. Median PFS for PDPN+ and PDPN− groups were 21.0 and 34.6 weeks, respectively (n=206, p=0.03). **(F)** Kaplan–Meier curves show that PDPN protein expression inversely correlates with OS among grade II/III diffuse astrocytoma patients (n=93, p=0.003). **(G)** Radiation response is correlated with PDPN expression (n=16, *p=0.0326). **(H)** MRI examples of a radiation responder **(A, B)** and non-responder **(C, D)**, pre- and post-radiation (RT).

RNA-Seq by Expectation–Maximization (RSEM) normalized (14) TCGA RNA sequencing and associated clinical data were assembled using the Broad Firehose data portal including all available samples from GBM (n=154) and lower grade glioma (n=513) cohorts. Isocitrate dehydrogenase (IDH) status for each of the samples was obtained from the LGG-GBM project (15). For PDPN-high and PDPN-low designations (**Figure 1B**), patients were placed into either category by splitting their mRNA expression levels into low or high categories (see **Supplementary Figure S1B**).

### Independent patient cohorts

Immunohistochemical (IHC) analysis of the prognostic significance of PDPN in GBM was performed on a group of patients for whom sufficient formalin-fixed and paraffin-embedded (FFPE) tissue remained from specimens previously examined for expression of proteins in the RAS signaling pathway and for the secreted protein YKL-40 (16, 17) in addition to a group of patients not previously published. Tumor samples used for real-time reverse-transcription polymerase chain reaction (qRT-PCR) analysis were selected from either the previously reported cohorts (with substantial overlap with the cases used for IHC in the current report) or from the unpublished group (with a single exception that was not in either group but for which total RNA was available). Details regarding the WHO grade II and III astrocytomas were reported previously (18, 19). All patients underwent pathological review to confirm diagnosis and usability of FFPE tissue for IHC.

### Determination of radiation therapy response

Determination of radiation therapy (RT) response was performed by comparing the enhancing tumor size between the postoperative magnetic resonance imaging (MRI) and the first post-RT MRI in the manner previously reported (20). RT response scores were determined for the majority of GBM patients who underwent subtotal tumor resection (STR) as determined by a ≤95% resection validated on postoperative MRI, as previously reported for the group of patients in the initial evaluation cohort (17).

### Immunohistochemistry

Immunohistochemistry (IHC) staining of FFPE tissue was performed as previously described (21). Details regarding the staining of MIB-1/Ki-67 and activated, phospho− intermediates (p−) of the ras signaling pathway, including p-Ser^473^ Akt (p-Akt) and p-Thr^202^/Tyr^204^ MAPK (p-MAPK), have been previously described (16, 18). Staining for PDPN was performed using the M2A monoclonal antibody (Vector Labs, Burlingame, CA). In short, slides were incubated in the primary antibody at an antibody dilution of 1:500 at 4°C overnight, and PDPN scoring was performed while blinded to clinical data using a two-tier system: negative (PDPN−), no tumor cell staining, and positive (PDPN+), detected in tumor cells in at least one medium power (100×) microscopic field. Cox multivariable analysis of GBMs was performed on negative vs. positive specimens.

### Real-time reverse transcription PCR

TaqMan qRT-PCR was performed as previously described (22) using TaqMan Reverse Transcription reagents (Life Technologies). Total RNA for tumor specimens was isolated using the MasterPure Complete RNA Purification kit (Epicentre Biotechnologies) according to the manufacturer’s instructions for paraffin-embedded tissue. Ten micrograms of total RNA was reverse transcribed with random hexamers. Quantitative RT-PCR was performed in triplicate using 100 ng cDNA in a 20-µl reaction containing 1× TaqMan Master Mix (Life Technologies), 800 nM each primer, and 250 nM of the appropriate Universal Probe (Roche Applied Science). Reactions were performed in a Chromo4 DNA Engine real-time thermal cycler (Bio-Rad) with the following cycling conditions: 95°C for 15 min for 1 cycle followed by 40 cycles of 95°C for 15 s and 60°C for 1 min. ΔC_T_ was calculated as the difference between the mean C_T_ of the tumor cDNA and the mean C_T_ of four reference genes (*GAPDH, RPLPO*, *TFRC*, and *GUSB*). Primer and probe combinations consisted of the following: *PDPN*, forward 5’GGGTCCTGGCAGAAGGAG3’, reverse 5’CGCCTTCCAAACCTGTAGTC3’, Universal Probe #20; *GAPDH*, forward 5’GGAAGCTTGTCATCAATGGAA3’, reverse 5’TTGATTTTGGAGGGATCTCG3’, Universal Probe #9; *TFRC*, forward 5’CCAGAGCTGCTGCAGAAAA3’, reverse 5’TGTTTTCCAGTCAGAGGGACA3’, Universal Probe #12; and *RPLPO*, forward 5’CTGGAAAACAACCCAGCTCT3’, reverse 5’GAACCAAAGCCCACATTCC3’, Universal Probe #74. *GUSB* amplification was performed using an Assays-on-Demand gene expression assay according to the manufacturer’s instructions (Life Technologies). For cell lines, 20-µ reaction mixtures were performed in triplicate and included 1 µl cDNA template and 1 µl each of primer and probe mix and TaqMan Universal PCR master mix (Life Technologies). Amplification proceeded as follows: denaturation at 95°C for 15 min and 40 cycles at 94°C for 1 min and 60°C for 1 min. ΔC_T_ was calculated as the difference between the mean C_T_ of the tumor cDNA and the mean C_T_ of the reference gene beta actin (*ACTB*). Primers and probes combinations consisted of *PDPN* #Hs01089983 and *ACTB* #Hs99999903 (Life Technologies).

### Cloning of *PDPN* knockdown constructs

PDPN shRNA (shLuc, MD5, and MD7) was cloned at Cellecta using the Cellecta pRSI-EF1a-TetRep-2A-Puro-H1Tet-(sh) vector cut at the BbsI restriction site. shRNA sequences were as follows: shLuc 5’-ACCGGCGCTGAGTACTTTGAAATGTTGTTAATATTCATAGCGACATTTCGAAGTACTCAGCGTTTT, MD5 5’-PDPN ACCGGGCTCCTCTTAAACATTTGTTGTGTTAATATTCATAGCACAGCAAATGTTTAGAGGAGCTTTT, MD7 5’-ACCGGCCAGGAGAGTAACAACTTAACGTTAATATTCATAGCGTTGAGTTGTTGCTCTCCCTGGTTTT. For miRNA experiments, miRNA was generated using the PDPN GenBank sequence NM_006474 in the Block-iT RNAi Designer (Life Technologies) to achieve the NM_006474_441_top 5’TGCTGACTTATAGCGGTCTTCGCTGGGTTTTGGCCACTGACTGACCCAGCGAACCGCTATAAGT3’ and NM_006474_441_bottom 5’CCTGACTTATAGCGGTTCGCTGGGTCAGTCAGTGGCCAAAACCCAGCGAAGACCGCTATAAGTC3’ miRNA oligo sequences, which were annealed and ligated into the Life Technologies Block-iT Pol II miR RNAi EmGFP expression vector per kit protocol (Life Technologies).

### Cell culture

The GSCs examined in this study have been published previously (23, 24). GSCs were cultured in Dulbecco’s modified Eagle’s medium (DMEM)-12 (1:1) with 1× B27 (Life Technologies), 20 ng/ml basic fibroblast growth factor (bFGF) (Sigma), 20 ng/ml epidermal growth factor (EGF) (Sigma), and 1% penicillin streptomycin solution (Cellgro). Human glioblastoma cell line U87 was obtained from American Type Culture Collection (ATCC) and maintained in DMEM containing 10% fetal bovine serum (FBS). NHA cells were generated and cultured as previously described (25). All cell cultures were grown at 37°C in a humidified atmosphere of 5% CO_2_.

### Generation of cell lines

For PDPN shRNA transduction experiments, GSCs were prepared as single-cell suspensions and seeded at 5×10^5^/well in a coated six-well plate on day 1. The cells were infected with lentivirus containing the shRNAs (MD1-MD12 and Luc) at multiplicity of infection (MOI)=5 in the presence of 0.4 µg/ml polybrene the following day. On day 3, the medium containing the lentivirus and polybrene was replaced with fresh regular medium to let the cells recover from infection. Finally, infected cells were cultured and expanded to T-25 and T-75 flasks. PDPN shRNAs identified as MD5 and MD7 was used in PDPN knockdown experiments. For experiments with adherent cell lines, U87 lines were grown to 80% confluence in 10-cm plates and transfected with 24 μg of appropriate vector containing either PDPN miRNA or corresponding empty vector (control), using Lipofectamine 2000 in Opti-MEM (Life Technologies). U87 transfectants were selected and maintained using blasticidin.

### Flow cytometry and FACS

FACSAria (BD Biosciences) was used to analyze and sort GSCs based on PDPN and CD133 expression using a PE-conjugated mAb to PDPN (clone NZ-1, AngioBio) alone or in combination with an APC-conjugated mAb to CD133 (clone 293C3, Miltenyi Biotech). Staining was performed per recommended manufacturer protocols.

### Orthotopic brain xenografts and *in vivo* imaging

For survival experiments, 2.5×10^4^ GSCs (sorted sub-populations) or 5×10^5^ GSCs (PDPN knockdown experiments) were directly injected into brains of athymic nude (nu/nu) mice of both sexes at age of 6–8 weeks using a stereotactic apparatus under anesthesia and with analgesics. Animals that showed signs of distress or were moribund were euthanized and autopsied. Doxycycline (Sigma) was administered *via* drinking water (2 mg/ml) containing 5% sucrose. Water was changed every 3 days. For *in vivo* bio-luminescent imaging, GSCs were engineered to express luciferase. On the day of imaging, animals were treated with luciferin (150 mg/kg, intraperitoneally). Tumor growth was monitored using IVIS 200 system bio-luminescent imaging, and tumor volume was measured using Living Image 4.7.3 software. All mice were cared for according to the guidelines and under the supervision of the Institutional Animal Care and Use Committee.

### Clonogenic survival assay

GSC neurosphere formation and radiation response was determined using the *in vitro* limiting dilution clonogenic survival assay (26). Prior to irradiation, cells were cultured in serum-free neurosphere medium for 5 days, dissociated into single-cell suspensions, and counted. Single-cell suspensions were then irradiated with various (2, 4, 6, and 8 Gy) doses. Irradiated single cells were plated in 200 µl of culture medium per well of 96-well round bottom plates. Each condition was plated in triplicate. Cells were incubated for 3–4 weeks at 37°C in 5% CO_2_ humidified incubators, and upon neurosphere formation, each well was examined for spheres and quantified. Plating efficiency (PE) values for treated cells were normalized to that of the control (non-irradiated) plates. The surviving fraction was determined by dividing the PE of treated cells by the PE of controls.

### Western blotting

Whole cell lysates were prepared from cultures using a standard NP-40 lysis buffer (Life Technologies) with 1× protease inhibitor tablet, 0.1 mM NaVO_3_, 1 mM dithiothreitol (DTT), and 1 µM phenylmethylsulfonyl fluoride (PMSF) (Roche Applied Science). Thirty micrograms of protein was loaded for sodium dodecyl sulfate–polyacrylamide gel electrophoresis (SDS-PAGE) using the standard protocols. Upon electrophoresis, protein was transferred onto a polyvinylidene fluoride (PVDF) membrane (Millipore) and incubated in 10% nonfat dry milk blocking solution for 30 min. Upon blocking, PVDF membranes were incubated overnight with primary monoclonal antibodies targeting PDPN (clone NZ-1, 1:200, AngioBio) and vinculin (1:1,000, Abcam) at 4°C. Membranes were subsequently washed and incubated with horseradish peroxidase (HRP)-conjugated anti-rat and anti-mouse secondary antibodies (Santa Cruz Biotechnology) at room temperature for 1 h and prepared for chemiluminescent detection using the ECL Plus Western Blotting Detection System kit (GE Healthcare Life Sciences) according to the manufacturer’s protocol.

### Transcriptome analysis

Total RNA was isolated from FACs-sorted PDPN+ and PDPN− subpopulations using the MasterPure Complete DNA/RNA Purification kit (Epicentre). The RNA samples were processed on Affymetrix U133A 2.0 microarray chips (Affymetrix). Raw microarray data were processed by affy (27) and limma (28) bioconductor packages using the custom CDF Brainarray EntrezG version 19 HGU133A2 (29). The heatmap was created using the heatmap.2 function in R. Significantly differentially expressed genes (absolute log2 fold change > 1 and p < 0.01) were clustered by hierarchical clustering using (1−Pearson correlation) as dissimilarity distance and complete method. Gene expression levels were normalized to Z-scores. Significantly up- and downregulated gene lists (p<0.05) were analyzed by Gene Set Enrichment Analysis (GSEA) (30) using the MSigDB C2 collection (31). GSEA default options were used, and enrichment was considered significant when significance was retained at twofold enrichment. The ENCODE ChIP-Seq Significance Tool was subsequently used to identify transcription factor binding sites within the significantly up- and downregulated gene lists (32). Five hundred base pairs up- and downstream represented padding sequences to enrich transcription factor binding. All data were deposited to the Gene Expression Omnibus (GEO series #GSE202221). Gene Ontology analysis was performed using the ShinyGO method with default settings (version 0.76) (33).

### Statistical analysis

For analysis of TCGA cohorts, *PDPN* expression grouped by IDH status or grade showed normally distributed strata, and p-values were subsequently obtained using two-sample Student’s t-test. Normalized read counts were log2 transformed and showed a bimodal distribution for *PDPN* expression. Clinical data consisted of tumor grade, histology, vital status, and follow-up time. Patients living at the time of this study had OS censored at the time of last follow-up. Nested models were compared using the likelihood ratio test (LRT). Analysis was conducted in R (v 3.1.2) using the survival package (34, 35). For analysis of the independent patient cohorts, primary clinical endpoints for analysis were OS, PFS, and RT response. Time to progression was determined from the date of surgery to the date of tumor recurrence or growth as first documented by MRI and confirmed in the clinical record. Univariate associations were determined by χ^2^ test or, when appropriate, the Fisher’s exact test (36) for categorical variables and the Wilcoxon rank sum test (37) or Student’s t-test, when appropriate, for associations with continuous variables. Subset analysis was performed as described previously (38). Recursive partitioning analysis was performed to determine the threshold for *PDPN* qRT-PCR data ablest to partition patients by vital status. All survival analysis was performed using the Kaplan–Meier method (39), and comparisons were made using the log-rank test. Multivariable analysis was performed using the Cox proportional hazards model (40) for survival or Spearman’s rank sum test (41). Analyses were performed in JMP Pro 12.1.0 (SAS, Cary, NC) and GraphPad Prism (GraphPad Software).

## Results

### PDPN expression is an independent prognostic marker in glioma

Elevated PDPN expression has been reported to correlate with short-term survival among malignant glioma patients (8, 42, 43). To further interrogate this association, we examined PDPN expression across 601 TCGA specimens with known IDH status (44–46). We found that PDPN expression is highly correlated with tumor grade (grade II, n=213; grade III, n=239; grade IV, n=149; p<0.0001; **Figure 1A**). When we compared IDH mutation status (a prognostic factor that correlates with better survival), we found that expression levels of PDPN are overall lower in grade I/II IDH-mutated glioma and are elevated in grade IV IDH-mutated specimens (IDH mutant, n=37; IDH wild-type, n=224; p<0.0001; **Supplementary Figure S1A**) (47). Others have now shown that the *PDPN* gene is amplified in a number of TCGA patient samples as well (15). PDPN expression exhibits a bimodal distribution, which, upon dichotomization (**Supplementary Figure S1B**), revealed a significant difference in patient survival (n=601, p<0.0001; **Figure 1B**).

We next examined PDPN protein expression by IHC in an independent GBM patient cohort (**Table 1**). PDPN staining was scored as either positive (PDPN+) or negative (PDPN−) (**Figure 1C**). IHC revealed 180 PDPN+ and 26 PDPN− cases. In PDPN+ cases, the protein characteristically stained within the cell cytoplasm and displayed increased staining on cell membranes. Vascular and perivascular cells did not stain, even in high-expressing tumors as has been described in other tissues (13). As identified in TCGA gene expression analysis (**Figure 1B**), PDPN protein expression was prognostic for GBM patients’ overall survival (OS) in our cohort presented here; median OS for PDPN+ and PDPN− groups were 53.4 and 148.1 weeks, respectively (p<0.0001; **Figure 1D**). PDPN protein expression was also prognostic for patient progression-free survival (PFS); median PFS was 21.0 weeks for the PDPN+ group and 34.6 weeks for the PDPN− group (p=0.03; **Figure 1E**). We found that IHC results were in close agreement with mRNA levels for several tumors that were tested (**Supplementary Figure S1C**). We used recursive partitioning analysis to select a fold expression that best separated survivors from deceased (**Supplementary Figure S1D**). Quantitative RT-PCR further revealed that PDPN gene expression was an independent prognostic marker for patient OS in the cohort: median OS for *PDPN*-high (n=53) and *PDPN*-low (n=18) groups were 37.0 and 240.7 weeks, respectively (n=71, p=0.0009; **Supplementary Figure S1E**). Multivariable analysis identified PDPN expression as an independent predictor of both OS (HR, 2.5; 95% CI, 1.42–4.71, p=0.0008) and PFS (HR, 1.7; 95% CI, 1.02–3.15, p=0.0413), while MAPK and AKT pathway activation were not predictive of survival (**Table 2**). We performed additional analyses on TCGA cohorts to understand if PDPN expression was correlated with MGMT methylation or TP53 mutations and found no strong correlation with either alteration with respect to PDPN expression levels (**Supplementary Figures S2A,B**).

**Table 1:** GBM patient characteristics.

Variable	Patients (%)
Total	206
Median age, years	59
Number <50 years (%)	49 (24)
Number ≥50 years (%)	157 (76)
Median survival, weeks	
Overall	58
Progression-free	22
RT Response	
Responder	15 (19)
Stable	13 (16)
Progression	53 (65)
Surgical Resection	
Gross total	103 (50)
Sub-total	98 (48)
Biopsy only	2 (1)
Resection type unknown	3 (1)

**Table 2:** Cox multivariate survival analysis for GBM patients.

	Overall survival		Progression-free survival	
Variable	HR (95% CI)	p-value	HR (95% CI)	p-value
Age (≥50 vs. <50 years)	2.1 (1.39−3.43)	0.0004	1.0 (0.69−1.63)	0.838
Surgical resection (STR/biopsy vs. GTR)	1.2 (1.06−1.45)	0.0076	1.3 (1.05−1.50)	0.0095
PDPN (+ vs. −)	2.5 (1.42−4.71)	0.0008	1.7 (1.02−3.15)	0.0413
p-MAPK (low vs. high)	1.0 (0.75−1.20)	0.739	0.9 (0.66−1.09)	0.211
p-Akt (+ vs. −)	0.8 (0.43−1.81)	0.671	0.9 (0.47−1.89)	0.759

To validate the prognostic significance of PDPN across malignant gliomas, we examined a cohort of 93 WHO grade II/III diffuse astrocytomas (**Supplementary Table S1**). WHO grade II (low grade) accounted for 43 (46%) and WHO grade III (intermediate grade) accounted for 50 (54%) of the cases. PDPN expression was analyzed by IHC and revealed that 30 (70%) low-grade and 35 (70%) intermediate-grade cases were PDPN+. Like GBM, lower-grade glioma patient OS also inversely correlated with PDPN protein expression (p=0.003) (**Figure 1F**). Indeed, PDPN expression was an independent predictor of survival in this cohort (HR, 3.9; 95% CI, 1.44–13.69) after adjusting for age, grade, and proliferative (MIB-1) index (**Table 3**). Neither MIB-1 index at a threshold previously reported (18) nor WHO grade was an independent predictor of OS in the multivariable analysis.

**Table 3:** Cox multivariate survival analysis for diffuse astrocytoma patients.

	Overall survival	
Variable	HR (95% CI)	p-value
Age (≥50 vs. <50 years)	1.0 (1.01−1.08)	0.0173
WHO grade (III vs. II)	1.6 (0.67−4.14)	0.292
MIB-1 index (>4 vs. ≤4)	1.9 (0.82−5.08)	0.133
PDPN (+ vs. −)	3.9 (1.44−13.69)	0.0058

Within the same cohort of GBM patients for which we analyzed PDPN protein expression, 103 (50%) of patients underwent gross total resection (GTR) and 98 (48%) of patients underwent sub-total resection (STR) or biopsy only (**Table 1**). Other than PDPN expression, surgical resection was the only parameter we identified in Cox multivariable analysis that was an independent predictor of both OS (HR, 1.2; 95% CI, 1.06–1.45) and PFS (HR, 1.3; 95% CI, 1.05–1.50) (**Table 1**). While PDPN expression inversely correlated with OS in patients who received GTR (p=0.0003; **Supplementary Figure S1F**), PDPN expression surprisingly was not prognostic in patients who underwent STR/biopsy (p=0.0639; **Supplementary Figure S1G**). PDPN expression was highly prognostic throughout our analysis, so we speculated that STR/biopsy variability and subjectivity might influence outcome and that PDPN expression should be evaluated in combination with radiotherapy (RT) in the STR/biopsy cohort. Accordingly, we evaluated the 81 STR/biopsy patients who received RT, and 15 cases (19%) showed RT response as detected by a decrease in the size of enhancing portion of the tumor seen in the first post-radiation MRI compared to the postoperative MRI (**Table 1**). Subset analysis of patients further revealed that 29% of patients from the PDPN+ group responded to RT compared with 70% in the PDPN− group (n=16, p=0.0326; **Figures 1G, H**).

### PDPN marks an aggressive subpopulation of glioma stem cells

**Figure 2:**
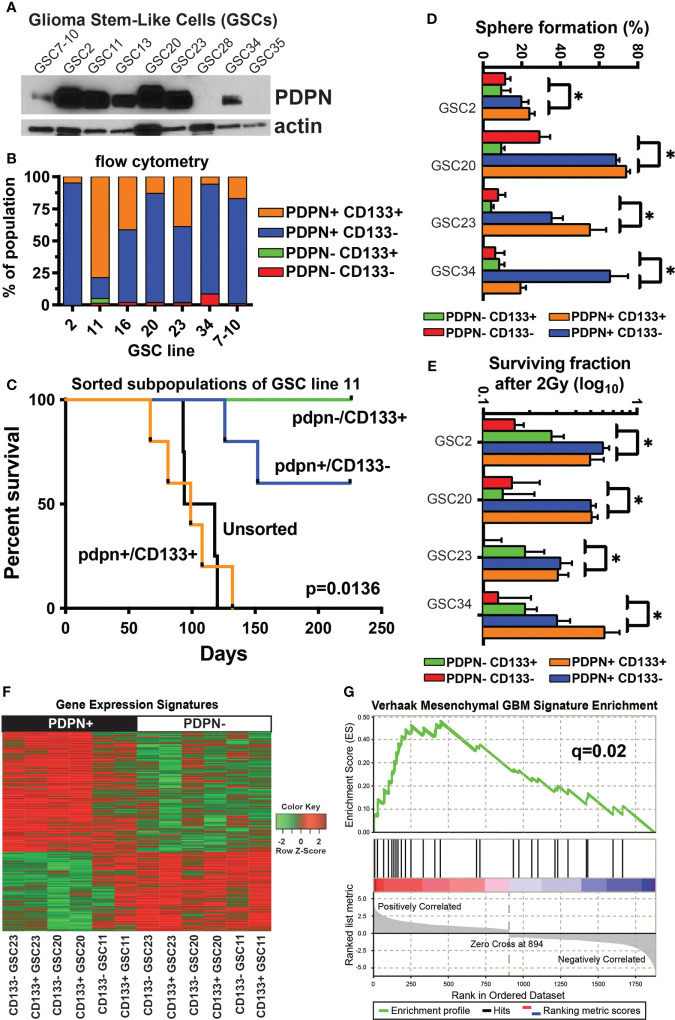
PDPN+ glioma-stem-like cells form aggressive tumors *in vivo* and mark a stem-like radioresistant subpopulation of cells. **(A)** PDPN protein is expressed in seven of the nine GSC lines shown. **(B)** Flow cytometry of GSCs shows PDPN expression is pervasive and more prominent in these GSCs. **(C)** Kaplan–Meier survival analysis of GSC11 PDPN−/CD133+, PDPN+/CD133+, and PDPN+/CD133− FACS-sorted subpopulations orthotopically injected into the brains of immunocompromised mice reveal that mice harboring tumors from CD133+/PDPN+ cells had a median survival of 99 days (n=5), while mice that received CD133+/PDPN− cells did not succumb to tumor formation (n=3) (p=0.0136, log-rank test). **(D)** PDPN+ FACS-sorted subpopulations have a higher sphere formation ability (*p<0.005, multiple t-tests, PDPN+ *vs*. PDPN− groups). **(E)** PDPN+ FACS-sorted subpopulations have a higher surviving fraction of cells after 2 Gy of radiation (*p<0.005, multiple t-tests, PDPN+ *vs*. PDPN− groups). **(F)** Transcriptome analysis revealed distinct gene expression signatures in PDPN+ and PDPN− sorted GSCs (three different cell lines). **(G)** GSEA of differentially expressed genes (p<0.05) demonstrated significant enrichment of the mesenchymal subtype signature (NES=2.04, q=0.02) in PDPN+ populations.

Since PDPN expression exhibited a prominent inverse correlation with glioma WHO grade, which are more anaplastic with increasing grade, we suspected that the glycoprotein may be involved with glioma differentiation. We investigated the potential relationship between cell membrane expression of PDPN and the putative cancer cell surface stem cell marker CD133. The CD133 protein is encoded by the *PROM1* gene and is present on leukemic and solid tumor cells (48). Both CD133+ (7, 49) and CD133− (50) GSCs have previously been identified in GBMs. Western blots of the majority of evaluated GSCs revealed high PDPN protein expression (**Figure 2A**). Using flow cytometry, we analyzed cultured GSCs for PDPN and CD133 cell surface expression (**Supplementary Figure S3A**). Interestingly, PDPN showed a more prominent representation on the cell surface than CD133 alone in GSC11 (**Figure 2B**). The majority of cells expressed PDPN (95%) and CD133 (82.0%). While a number of PDPN+ cells did not express CD133 (16.3%), most CD133+ cells co-expressed PDPN (78.7%) (**Figure 2B**). Indeed, while CD133 representation varied, PDPN was detected at consistently high levels, and nearly all cells that expressed CD133 also expressed PDPN (**Figure 2B**).

Based on the differential cell surface expression of PDPN and CD133, we isolated GSC subpopulations and evaluated their tumor-forming capacities. We sorted PDPN+CD133+ and PDPN-CD133+ subpopulations of GSC11 and orthotopically injected them into the brains of immunocompromised mice. GSC11 was selected because it had the most prominent PDPN-CD133+ population. Mice harboring PDPN+CD133+ tumors had a median survival of 99 (n=5) days, while mice that received PDPN-CD133+ cells did not succumb to tumor formation (n=3) (p=0.0136, log-rank test; **Figure 2C**).

Because PDPN+CD133+, but not PDPN-CD133+, GSCs efficiently formed tumors *in vivo*, we decided to further evaluate the tumorigenic potential of PDPN+ GSCs *in vitro*. We evaluated neurosphere formation of sorted GSC23 differentially expressing PDPN and CD133 using the *in vitro* limiting dilution clonogenic survival assay. PDPN+ GSCs produced significantly more neurospheres than PDPN− GSCs irrespective of CD133 surface expression (**Supplementary Figure S3B**). This same trend was observed across the cohort of tested GSCs (p<0.005, multiple t-tests, PDPN+ vs. PDPN− groups, **Figure 2D**). We further tested whether or not PDPN expression influences GSC neurosphere formation following irradiation *in vitro*. Significantly more PDPN+ neurospheres survived upon irradiation compared with PDPN− neurospheres (**Supplementary Figure S3C**). Notably, GSC survival was greater in the PDPN+ population upon irradiation than in the unsorted population. CD133 yielded no survival advantage to irradiated PDPN+ GSCs, and similar results were observed across the cohort of GSCs tested (p<0.005, multiple t-tests, PDPN+ vs. PDPN− groups, **Figure 2E**).

We next investigated the mechanism of PDPN-associated RT resistance in GBM by whole transcriptome analysis on a panel of GSCs differentially sorted based on PDPN and CD133 cell surface expression. Differential expression analysis (p<0.05) revealed sharply contrasting gene expression signatures in PDPN+ and PDPN− GSCs (**Figure 2F**; **Supplementary Tables 2, 3**). PDPN has previously been correlated with the mesenchymal GBM subtype (43), and Verhaak et al. identified an inverse correlation between PDPN expression and the proneural subtype. Given this, we performed GSEA (30) of PDPN+ GSCs and found enrichment of the mesenchymal subtype signature (NES=2.04, q=0.02; **Figure 2G**), which further validated the findings that PDPN+ cells are associated with a more aggressive phenotype then PDPN− subpopulations. Gene Ontology analysis revealed a number of processes enriched in the up- or downregulated differentially regulated genes (**Supplementary Tables 4, 5**). Notably, a number of gene groups that are downregulated in PDPN+ cells are involved in cellular differentiation.

### PDPN is associated with aggressive tumor characteristics and radioresistance

**Figure 3:**
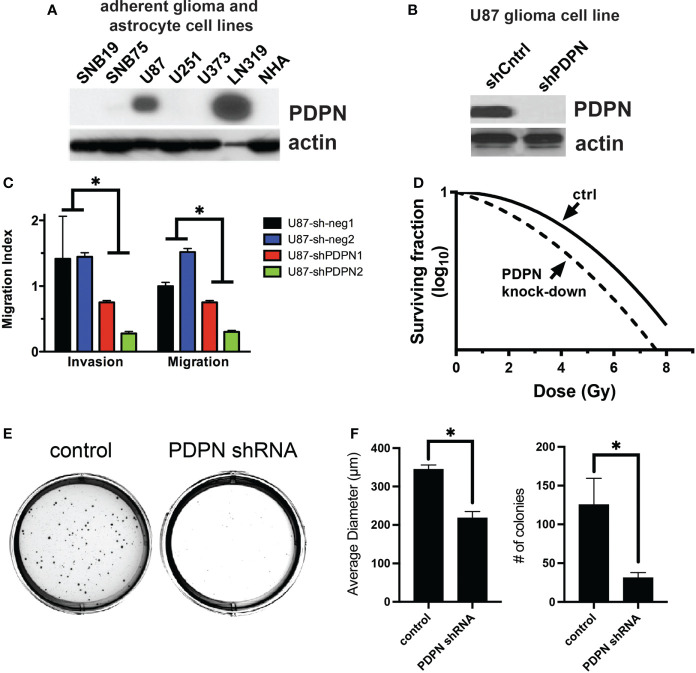
PDPN knockdown sensitizes adherent glioma cell lines to radiation and reduces invasion, migration, and colony formation ability. **(A)** PDPN expression in adherent glioma cell lines and normal human astrocytes (NHA). **(B)** Western blot of PDPN knockdown in U87 cell lines. **(C)** Invasion and migration assays of U87 PDPN knockdown lines (*p<0.005, multiple t-tests, sh-neg *vs*. sh-PDPN). **(D)** Clonogenic radiation survival assay with PDPN knockdown lines, PDPN silencing sensitized U87 cells to *in vitro* radiation compared with controls. **(E)** Representative crystal violet stains after a colony formation assay with U87 PDPN knockdown lines. **(F)** Quantification of colony diameter and colony number after colony formation assay of control and PDPN shRNA cell lines (*p<0.005, two tailed t-test).

To understand if PDPN may be playing a role in tumor aggressiveness and radioresistance in additional cell lines and contexts, we performed a series of experiments using adherent cell lines, which are grown in serum (unlike GSCs, which are grown in serum-free conditions). We first tested for PDPN expression in multiple adherent human glioma cell lines, using normal human astrocytes (NHA) as a negative control and found that PDPN was highly expressed in U87 and LN319 cells (**Figure 3A**). We created U87 PDPN knockdown lines (**Figure 3B**). Using invasion and migration assays, we found that PDPN knockdown led to reduced invasion and migration (p<0.005, multiple t-tests, sh-neg vs. sh-PDPN, **Figure 3C**). To test for the association with radioresistance, we performed a clonogenic survival assay with 0, 2, 4, 6, and 8 Gy of radiation and found a significant decrease in the surviving fraction of cells at all dose levels (**Figure 3D**). Colony formation assays showed a significant decrease in both the number and size of colonies formed when PDPN was knocked down (p<0.005, two-tailed t-test, **Figures 3E, F**).

### Knockdown of PDPN in glioma-stem-like cells slows intracranial tumor growth and extends overall survival time in mice

**Figure 4:**
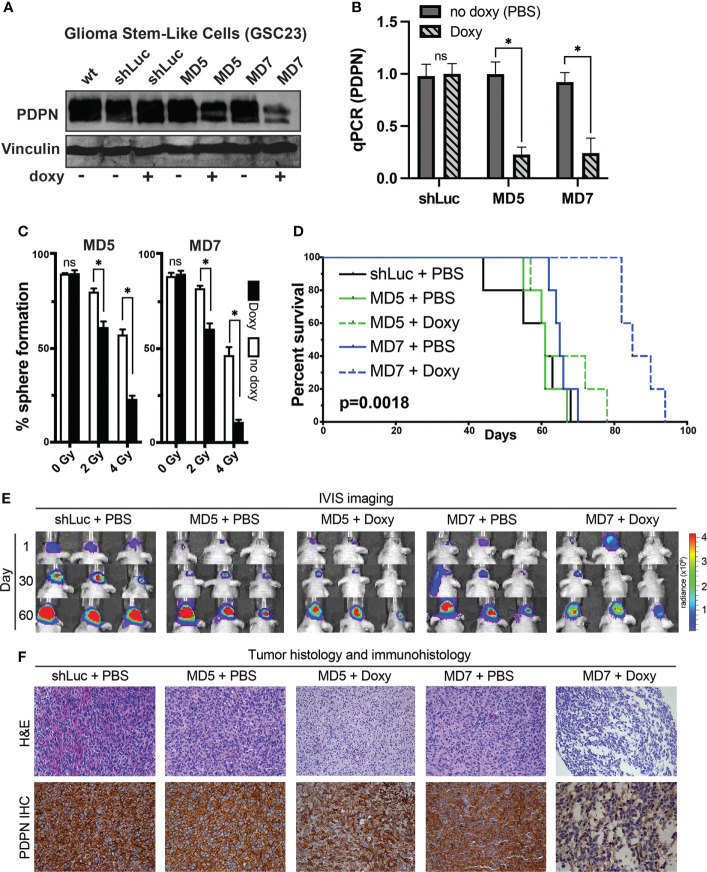
PDPN knockdown in glioma stem-like cells leads to radiosensitization *in vitro* and slows intracranial tumor growth. **(A)** Western blot of GSC line 23 (GSC23) showing PDPN expression levels after exposure to doxycycline (doxy), indicating inducible PDPN knockdown. **(B)** qPCR validation of PDPN knockdown (*p<0.005, two-tailed t-test). **(C)** *In vitro* clonogenic survival assays revealed that PDPN silencing by the shRNA MD5 and MD7 was effective in sensitizing GSC23 spheres to radiation in a dose-dependent manner [0 Gy, not significant (ns); 2 Gy, *p<0.0001; 4 Gy, *p<0.0001]. **(D)** Kaplan–Meier survival analysis (n=5 per condition) of PDPN knockdown, with negative (shLuc and no doxy) controls (p=0.0018, log-rank test). **(E)** Time course IVIS imaging of tumors from days 1, 30 and 60. **(F)** Representative tumor histology and PDPN IHC of animal tumors at time of animal death.

To understand how PDPN may alter intracranial xenograft growth, we first attempted to generate PDPN knockout lines. We found that whole-culture PDPN knockout lines were not viable in two different GSC lines with four different CRISPR guide RNAs targeting exons 2 or 3 (data not shown). Single-colony selection or sorting of PDPN− cells from knockout lines were not performed, as GSCs are very heterogeneous and have highly variable intra-cellular growth rates and characteristics (as shown in **Figure 2**). This is in contrast to a recent report wherein PDPN was knocked out successfully but did not affect tumor growth characteristics (51). This discrepancy may be due to the selection method used by the authors, who sorted for a PDPN− population after performing knockout, which may have artificially selected for a propagating PDPN− sub-population after PDPN KO selection, making comparisons to control populations difficult. To circumvent these issues, we created doxycycline-inducible PDPN knockdown lines, which would theoretically more closely mimic drug inhibition and could be compared to no-doxycycline controls. Interestingly, generating stable knockdown GSC lines of PDPN was challenging, and 12 different shRNAs had to be tested (data not shown), which yielded two knockdown lines termed MD5 and MD7, as confirmed by Western blot (**Figure 4A**). We confirmed that doxycycline appropriately led to the decrease in PDPN mRNA (p<0.005, two-tailed t-test, **Figure 4B**). Knockdown of PDPN using the MD5 and MD7 line was sufficient to sensitize GSCs to 2 or 4 Gy of radiation, as assessed by sphere formation assay [0 Gy, not significant (ns); 2 Gy, p<0.0001; 4 Gy, p<0.0001; **Figure 4C**].

To understand if induction of PDPN knockdown after intracranial injection could slow tumor growth *in vivo*, we fed animals doxycycline after implantation and for the duration of the study (n=5 mice per condition, **Figure 4D**). Indeed, silencing PDPN significantly slowed the ability of GSCs to form tumors compared with controls. Overall median survival times were 8.7 weeks for control, 8.7 weeks for MD5 + PBS, 9.5 weeks for MD5 + doxy, 9.3 weeks for MD7 + PBS, and 12.1 weeks for MD7 + doxy (p=0.0018, log-rank test). We tracked tumor bioluminescence over days 1, 30, and 60, using IVIS imaging (**Figure 4E**), which corroborated these findings. We further found that these tumors appeared morphologically similar as assessed by H&E and that PDPN knockdown was maintained (as verified qualitatively by IHC) (**Figure 4F**).

## Discussion

The mucin-type transmembrane glycoprotein PDPN is important for the development of multiple organs, and expression of PDPN in a large number of human tumors suggests that the protein may have a functional role in tumor development or progression (52). Mishima et al. identified increasing PDPN expression across malignant astrocytic tumors (13), which was later attributed to aberrant PI3K-AKT-AP1 signaling pathway regulation (53). In the present study, we demonstrated that PDPN is an independent prognostic marker of patient survival in glioma. Our results corroborate with the work that previously associated PDPN expression with patient survival (8, 51), and we have effectively overcome limitations reported in a recent study that did not find PDPN expression to be prognostic in GBM (54).

We explored the prognostic value of *PDPN* expression across gliomas in TCGA and verified the findings in independent glioma cohorts at both the protein and mRNA levels. The absence of PDPN identified a subset of GBM patients who had a median survival of nearly 3 years (148.1 weeks). The prognostic relevance of PDPN was independent of both extent of surgical resection and of age, which is one of the strongest predictors of outcome for GBM patients. Furthermore, PDPN expression increased from 70% in low-intermediate-grade astrocytomas to 87% in high-grade astrocytomas (GBMs). Mishima et al. found PDPN expression to be absent in WHO grade II tumors while present in 36% of WHO grade III and 53% of WHO grade IV tumors (13). The numerical discordance between our results and those of Mishima et al. might be due to the different monoclonal antibodies used for IHC detection. This may be particularly true with respect to the low-grade diffuse astrocytoma analysis, as PDPN mRNA was detected by qRT-PCR in the low-grade tumors in that study. Another potential reason for discordance is the subjectivity among pathologists in distinguishing grade II from grade III astrocytomas.

Interestingly, in accordance with Mishima et al., we did not observe PDPN+ GSCs around the perivascular niche. This suggests that perivascular GSCs may contain PDPN-negative cells, which implies that PDPN does not ubiquitously mark all stem cell populations found in GBM. Therefore, it should be noted that PDPN may not serve as a ubiquitous GSC marker according to our results. Indeed, there are many different cancer-stem-cell niches that exist within the glioma environment (i.e., perivascular, hypoxic, invasive, tumor border, white matter, and necrotic niches), and the role of PDPN and other cellular markers within those niches remains to be defined (55–58). More specifically, given this apparent lack of distribution of PDPN around the perivascular niche, future studies may aim to understand how oxygen tension, or hypoxic conditions, may regulate PDPN biology in glioma.

To explore the biological role of PDPN, we studied its function in human adherent glioma cell lines and patient-derived glioma-stem-like cultures. Consistent with our findings implicating PDPN in glioma malignancy and an association with a mesenchymal phenotype, a model for PDPN in tumor invasion has been proposed in epithelial tumors whereby PDPN was shown to redistribute the membrane cytoskeleton linker ezrin to filopodia-like structures and reduce cell–cell adhesiveness (59). *PDPN* silencing has been shown to reduce the invasive capacity of GBM cells (8), and combined evidence supports further investigation of the role of PDPN in GBM cell migration and invasion. Wicki et al. showed that PDPN promotes tumor cell migration by filopodia in the absence of epithelial–mesenchymal transition (EMT) (60). Intriguingly, IHC staining revealed co-expression of PDPN and E-cadherin at the invasive front. In contrast, using a different model, Martin-Villar et al. showed complete EMT in which PDPN expression induced a classic E- to N-cadherin switch (10).

We further tested the role of PDPN in radioresistance and found that knockdown of PDPN was sufficient to sensitize glioma cell lines to radiation. This was consistent with our finding that patients with low levels of PDPN have a much higher likelihood to respond to radiotherapy treatment. This suggests that PDPN may serve as a predictive marker to radiotherapy in patients, in addition to its putative prognostic value. To understand if PDPN targeting may have therapeutic potential, we performed a series of *in vivo* intracranial experiments with inducible PDPN-knockdown GSC lines. Our findings demonstrate reduced growth rates of tumors with PDPN knockdown, and we observed extended survival times in mice. These results are in contrast to reports wherein PDPN was knocked out *via* CRISPR-Cas9 and sorted for PDPN-negative populations that continue to divide in culture, making control comparisons difficult to establish (51).

In conclusion, PDPN marks an aggressive sub-population of GSCs that exhibit increased treatment resistance. Our work suggests that targeting of PDPN may be a therapeutic option in glioblastoma. Future studies may include understanding the molecular mechanisms of PDPN’s molecular function in glioblastoma and its interaction with the microenvironment, validating its prognostic role as a clinical biomarker, and further characterizing its potential as a therapeutic target.

## Data availability statement

The datasets presented in this study can be found in online repositories. The names of the repository/repositories and accession number(s) can be found in the article/**Supplementary Material**.

## Ethics statement

The animal study was reviewed and approved by NYU School of Medicine Animal Ethics Committee. Written informed consent was not obtained from the individual(s) for the publication of any potentially identifiable images or data included in this article.

## Author contributions

EE, QW, ES, RV, RE, JG, LG, TL, KB, LL, ZY, and YD conducted the experiments. AM, EE, QW, ES, RV, FL, and KA conceptualized the experimental design and methodology. AM, EE, QW, ES, RE, and RV performed the analyses. AM, EE, QW, ES, and RV performed the writing, reviewing, and editing. FL and KA provided resources. All authors contributed to the article and approved the submitted version.

## Funding

This work was supported by the NIH/NCI under award numbers R01CA190121 (ES, RV), P50CA127001 (ES, RV, KA, FL), P01CA085878 (RV), and P30CA016672 Cancer Center Support Grant (flow cytometry and cellular imaging, the research animal support, and sequencing and microarray facilities); by the American Brain Tumor Association (ES); by the National Brain Tumor Society Defeat GBM Research Collaborative (ES, RV, FL); by the Cancer Prevention and Research Institute of Texas under award number RP120256 (ES, FL, RV) and RP140606 (RV); and by the Broach Foundation (FL)

## Conflict of interest

The authors declare that the research was conducted in the absence of any commercial or financial relationships that could be construed as a potential conflict of interest.

## Publisher’s note

All claims expressed in this article are solely those of the authors and do not necessarily represent those of their affiliated organizations, or those of the publisher, the editors and the reviewers. Any product that may be evaluated in this article, or claim that may be made by its manufacturer, is not guaranteed or endorsed by the publisher.
