# Supplementary material for: PDPN marks a subset of aggressive and radiation-resistant glioblastoma cells
Source: Front Oncol. 2022 Aug 10;12:941657. doi: 10.3389/fonc.2022.941657 (PMC9434399; doi:10.3389/fonc.2022.941657)
Supplement: Supplementary file 1 [file DataSheet_1.pdf]

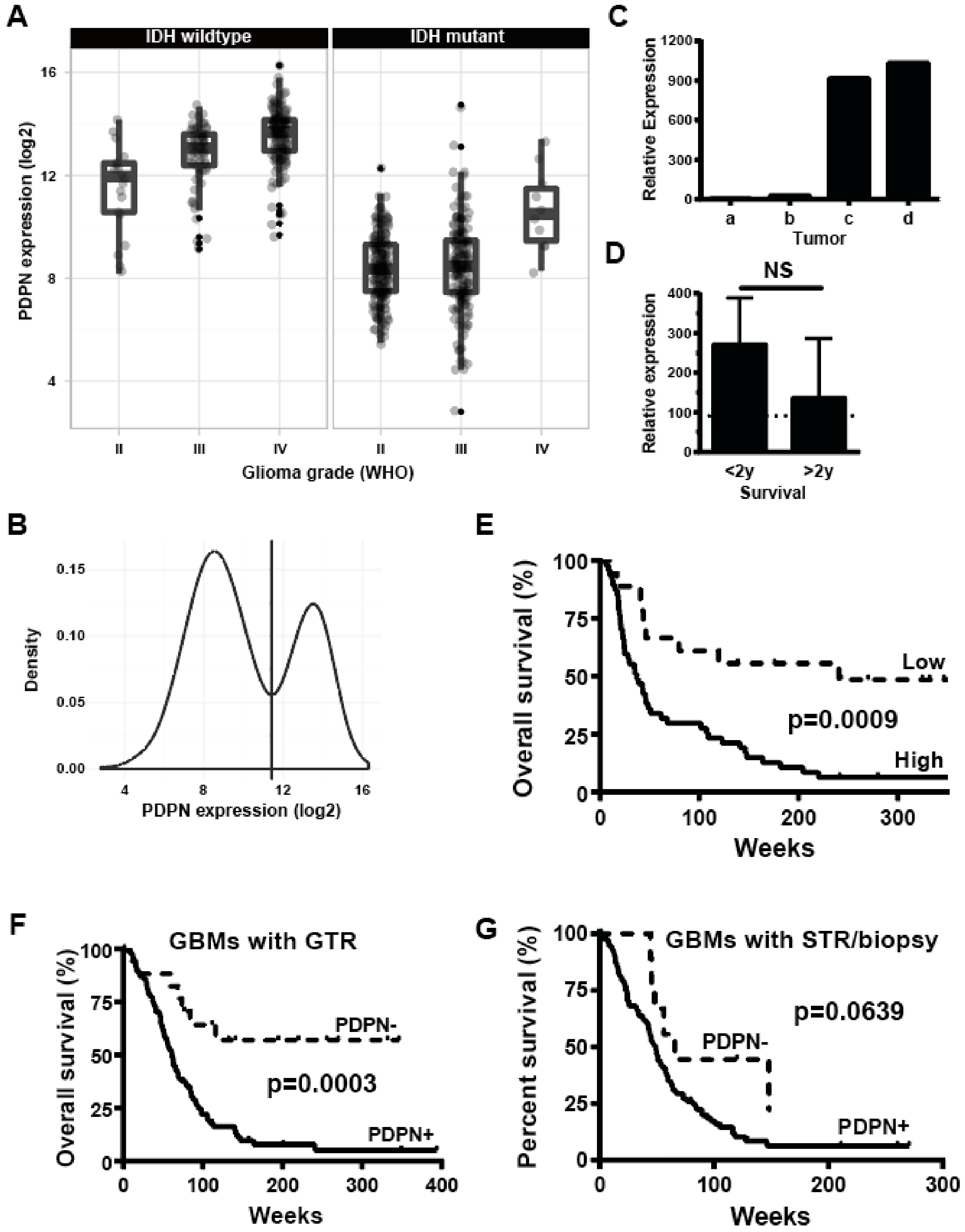

Supplementary Figure 1. **(A)** Boxplots show increasing PDPN expression across increasing glioma grades categorized by IDH mutational status. IDH wild-type tumors show 3-fold higher PDPN expression ($p<0.0001$) compared with IDH mutant tumors, and grade IV tumors show 2-fold increased PDPN expression relative to grade II tumors ($p<0.0001$). **(B)** PDPN expression exhibits a bimodal distribution across glioma grades in TCGA. Vertical line corresponds with a dichotomization value of 11.4. **(C)** PDPN was measured by QRT-PCR following reverse transcription of total RNA from FFPE tissues. **(D)** Recursive partitioning analysis was used to select a fold-expression that best separated survivors from deceased (dashed line). **(E)** Kaplan-Meier curves

show that PDPN expression inversely correlates with OS among GBM patients. Median OS for PDPN-high and PDPN-low groups was 37.0 and 240.7 weeks, respectively (n=71, p=0.0009, log-rank test). **(F)** PDPN expression inversely correlated with OS in patients who received GTR (p=0.0003, log-rank test). **(G)** Kaplan-Meier curves show that PDPN protein expression did not significantly influence OS of patients receiving STR/biopsy in our analysis (p=0.0639, log-rank test).

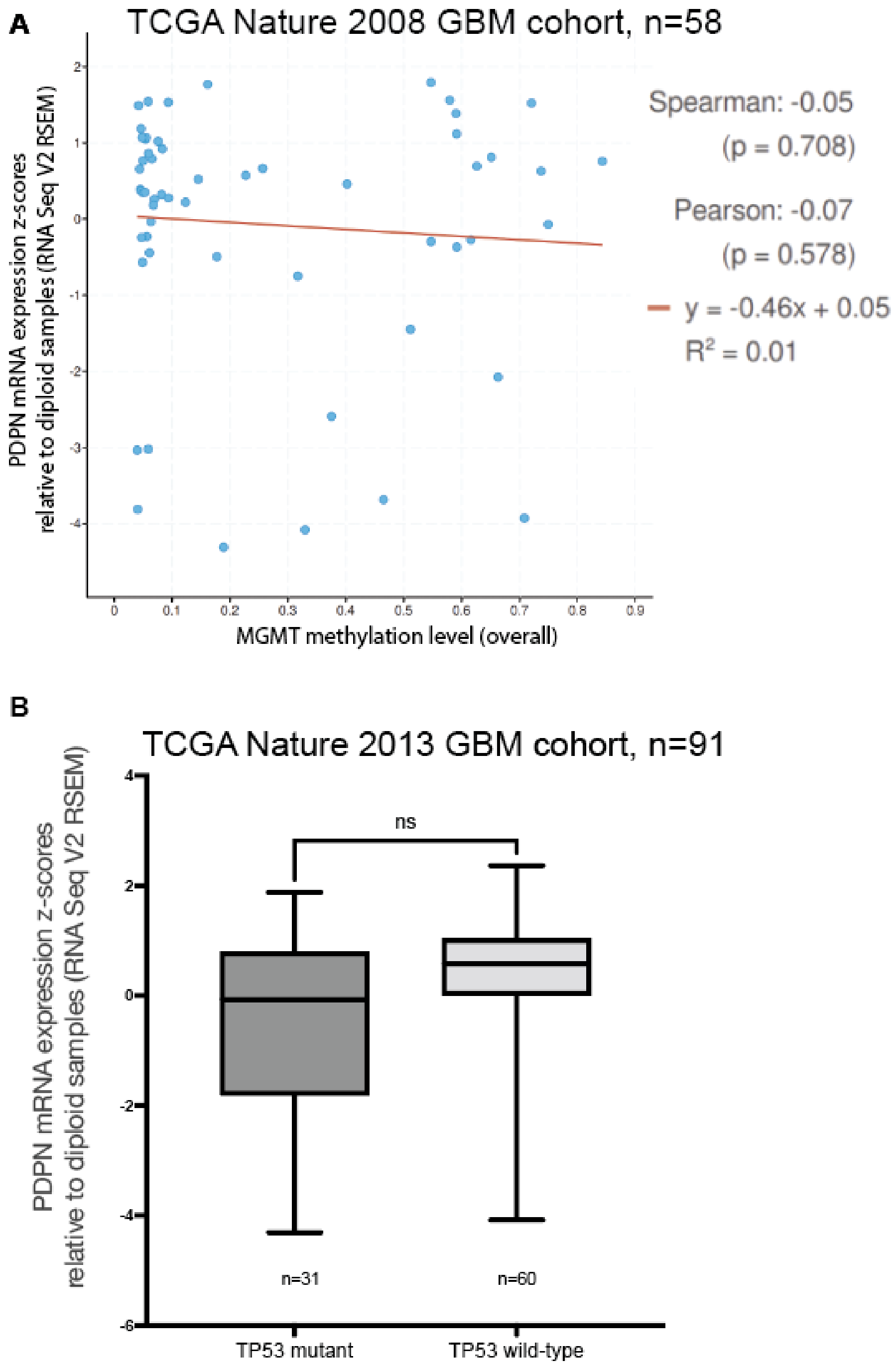

Supplementary Figure 2. (A) PDPN mRNA expression from TCGA Nature 2008 GBM cohorts plotted against overall MGMT methylation level show no strong correlation. **(B)** PDPN mRNA expression from TCGA Nature 2013 GBM cohort plotted by TP53 status shows no significant difference between the two groups (two-tailed t-test, not significant).

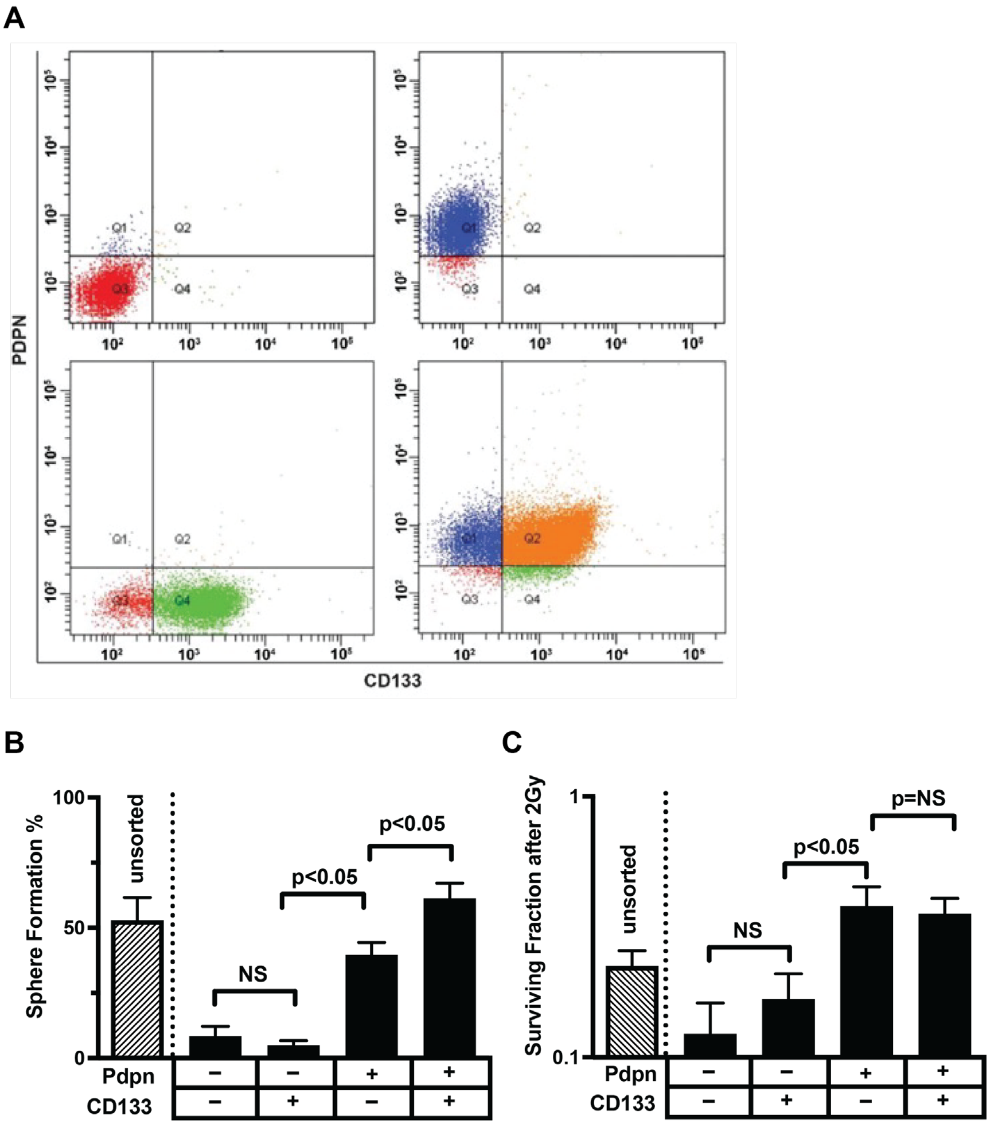

Supplementary Figure 3. (A) Flow cytometry example of GSC11 using a PE-conjugated mAb to PDPN (clone NZ-1, AngioBio) alone or together with an APC-conjugated mAb to CD133 (clone 293C3, Miltenyi Biotech). Cells were incubated with PE and APC fluorophores alone (upper left), PE-conjugated mAb to PDPN (upper right), APC-conjugated mAb to CD133 (lower left), and PE-conjugated mAb to PDPN together with APC-conjugated mAb to CD133 (lower right). The majority of cells expressed PDPN (95%) and CD133 (82.0%). While a number of PDPN+ cells did not express CD133 723 (16.3%), most CD133+ cells co-expressed PDPN (78.7%). (**B**) Sphere formation percentage and (**C**) surviving fraction of cells after 2 Gy of radiation of GSC line 23 on sorted populations (two-tailed t-test).

A

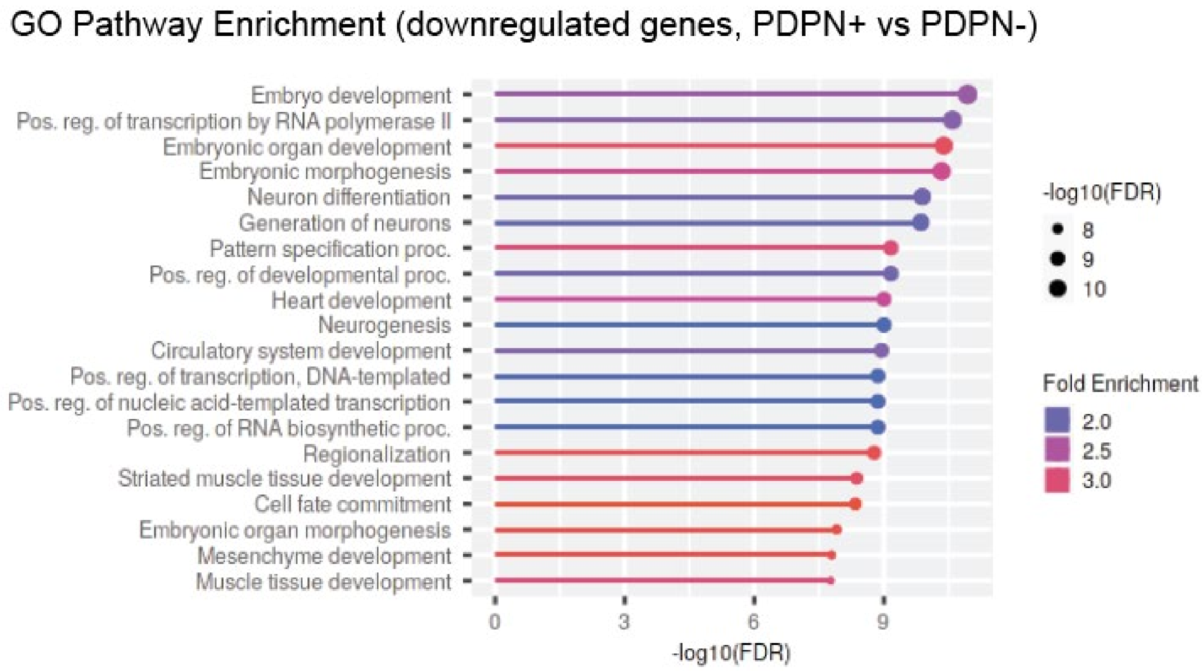

B

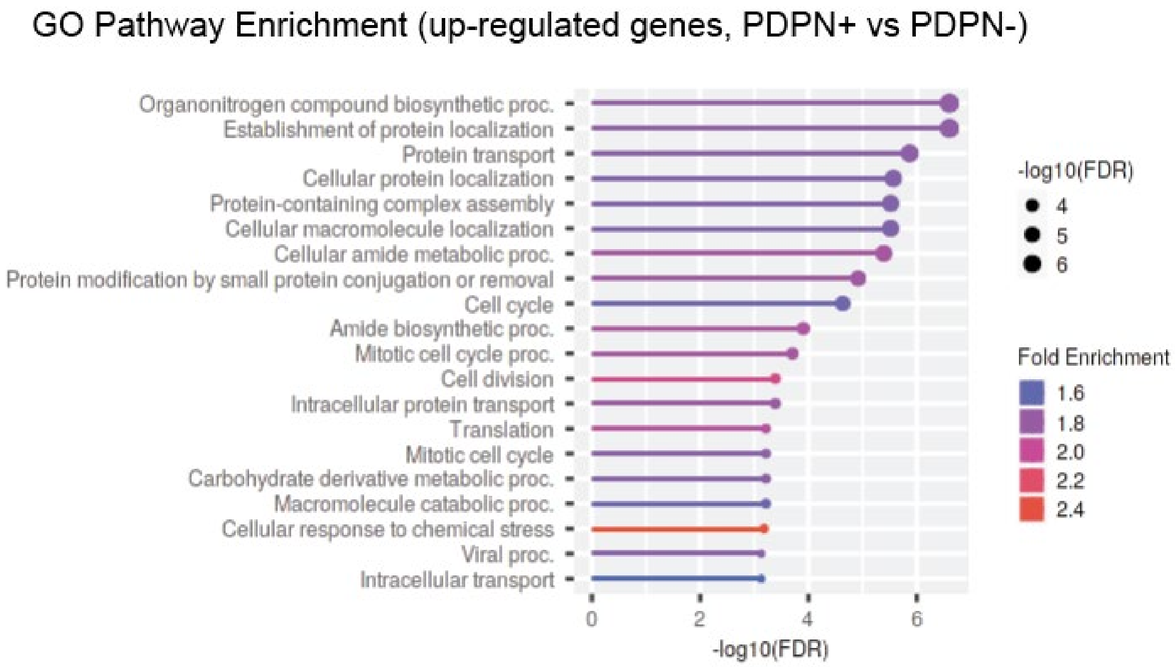

Supplementary Figure 4. GO pathway enrichment of down-regulated (A) and up-regulated (B) genes from PDPN+ and PDPN- subpopulations of GSCs.

Supplementary Table 1.

Table S1. Diffuse astrocytoma patient characteristics

Variable	Patients (%)
Total	93
Median age, years	36
Number <50y (%)	77 (83)
Number ≥50y (%)	16 (717)
Median survival, months	
Overall	151
WHO grade	
II	43 (46)
III	50 (54)
